# Comparison of Pathologic Response Evaluation Systems after Anthracycline with/without Taxane-Based Neoadjuvant Chemotherapy among Different Subtypes of Breast Cancers

**DOI:** 10.1371/journal.pone.0137885

**Published:** 2015-09-22

**Authors:** Hee Jin Lee, In Ah Park, In Hye Song, Sung-Bae Kim, Kyung Hae Jung, Jin-Hee Ahn, Sei-Hyun Ahn, Hak Hee Kim, Gyungyub Gong

**Affiliations:** 1 Department of Pathology, University of Ulsan College of Medicine, Asan Medical Center, Seoul, Korea; 2 Department of Oncology, University of Ulsan College of Medicine, Asan Medical Center, Seoul, Korea; 3 Department of Surgery, University of Ulsan College of Medicine, Asan Medical Center, Seoul, Korea; 4 Department of Radiology, University of Ulsan College of Medicine, Asan Medical Center, Seoul, Korea; University of North Carolina School of Medicine, UNITED STATES

## Abstract

**Purpose:**

Several methods are used to assess the pathologic response of breast cancer after neoadjuvant chemotherapy (NAC) to predict clinical outcome. However, the clinical utility of these systems for each molecular subtype of breast cancer is unclear. Therefore, we applied six pathologic response assessment systems to specific subtypes of breast cancer and compared the results.

**Patients and Methods:**

Five hundred and eighty eight breast cancer patients treated with anthracycline with/without taxane-based NAC were retrospectively analyzed, and the ypTNM stage, residual cancer burden (RCB), residual disease in breast and nodes (RDBN), tumor response ratio, Sataloff’s classification, and Miller—Payne grading system were evaluated. The results obtained for each assessment system were analyzed in terms of patient survival.

**Results:**

In triple-negative tumors, all systems were significantly associated with disease-free survival and Kaplan-Meier survival curves for disease-free survival were clearly separated by all assessment methods. For HR+/HER2- tumors, systems assessing the residual tumor (ypTNM stage, RCB, and RDBN) had prognostic significance. However, for HER2+ tumors, the association between patient survival and the pathologic response assessment results varied according to the system used, and none resulted in distinct Kaplan—Meier curves.

**Conclusion:**

Most of the currently available pathologic assessment systems used after anthracycline with/without taxane-based NAC effectively classified triple-negative breast cancers into groups showing different prognoses. The pathologic assessment systems evaluating residual tumors only also had prognostic significance in HR+/HER2- tumors. However, new assessment methods are required to effectively evaluate the pathologic response of HR+/HER2+ and HR-/HER2+ tumors to anthracycline with/without taxane-based NAC.

## Introduction

Neoadjuvant chemotherapy (NAC) is often used to treat three categories of patient: those with locally advanced breast cancer; those with operable breast cancer who are not candidates for breast-conserving surgery; and those with proven lymph node metastases [1, 2]. NAC induces a spectrum of morphologic changes in tumors and lymph nodes, including the complete disappearance of invasive cancer cells (pathologic complete response [pCR]), partial tumor regression, no response, or progressive tumor growth during treatment [3–5]. The pCR rate varies according to the molecular subtype of breast cancer and the therapeutic regimen [6, 7], and correlates well with prolonged survival [7, 8]. However, the majority of post-NAC breast cancer cases show residual tumor in the tumor bed.

Several pathologic response evaluation systems for residual cancer have been proposed. These evaluation systems can be roughly divided into two categories: absolute assessment of the residual tumor and relative assessment of the treatment response (comparing the cellularity or tumor size of post-NAC specimens with those of pre-NAC specimens or images)[9–14]. Parameters such as ypTNM stage, residual disease in breast and nodes (RDBN), and residual cancer burden (RCB) evaluate only residual tumor in the breast parenchyma and lymph nodes [6, 13, 15]. Conversely, Miller—Payne grading and Sataloff’s classification compare the size and cellularity of the pre- and post-NAC tumor [9, 10]. The recently developed tumor response ratio (TRR) compares tumor size on pre-NAC images and post-NAC microscopic tumor size [14]. Each evaluation system predicts survival outcome for breast cancer patients. Recent studies compared several of these classification systems and found that they yielded different predictive values.[16, 17] However, no standardized and/or superior pathologic response evaluation system exists at the present time.

Breast cancers can be classified using immunohistochemistry-based approaches, the results of which correlate well with the molecular subtypes determined by microarray-based analyses of intrinsic gene expression [18]. For example, the luminal A subtype is estrogen receptor(ER)-positive, progesterone receptor(PR)-positive, and human epidermal growth factor receptor (HER)2-negative (ER+/PR+/HER2-); the luminal B subtype is ER+/PR+/HER2+; the HER2-positive subtype is ER-/PR-/HER2+; and the triple-negative subtype is ER-/PR-/HER2-. These molecular classifications have some prognostic value [19, 20]. Previously, we revealed that each subtype of breast cancer shows intrinsic morphologic differences and characteristic pathologic response patterns to anthracycline and taxane-based NAC [21]. Triple-negative tumors frequently presented as a single mass on pre-NAC MRI analyses, and pre-NAC biopsy specimens showed high overall and invasive cancer cellularity. Hormone receptor (HR)- tumors showed higher nuclear and histologic grades, and denser lymphocytic infiltration than HR+ tumors. The tumors within each subtype retained their morphologic features after NAC. For example, pushing margins, high grade, and high cellularity were observed in triple-negative breast cancers, whereas an infiltrative growth pattern and abundant *in situ* components were observed in HR+ subtypes. These differences might affect the classification of residual tumors according to different pathologic evaluation systems. Therefore, the most effective system for evaluating the NAC response might be different for each subtype of breast cancer. However, no studies have compared different pathologic evaluation systems for each subtype of breast cancer.

Therefore, the aims of this study were to compare pathologic response assessment systems and identify the one that is best for predicting outcome in patients with different subtypes of breast cancer.

## Materials and Methods

### Patients and treatments

In total, 588 female patients were diagnosed with primary breast cancer by core needle biopsy, and all underwent anthracycline with/without taxane-based NAC, followed by definitive surgical excision at Asan Medical Center (Korea) from 2010 to 2012. The patient group yielded 594 tumor specimens (the group included six cases of bilateral breast cancer). The NAC regimen, either anthracycline alone or anthracycline plus taxane, was determined according to the involvement of axillary lymph nodes. None of the patients received neoadjuvant trastuzumab. All patients underwent dynamic contrast-enhanced breast MRI before NAC to measure the number of masses and to determine tumor size.

Of the 588 patients included in the study, 147 (25%) received an anthracycline-based NAC regimen and 441 (75%) received an anthracycline and taxane-based NAC regimen. Anthracycline-based regimens included three to five cycles of 60 mg/m^2^ adriamycin and 600 mg/m^2^ cyclophosphamide. Anthracycline and taxane-based regimens included either four cycles of 75 mg/m^2^ docetaxel plus 50 mg/m^2^ adriamycin, or four cycles of 60 mg/m^2^ adriamycin and 600 mg/m^2^ cyclophosphamide followed by four cycles of 75 mg/m^2^ docetaxel. Surgery was performed approximately 3–4 weeks after the final chemotherapy cycle. This study was conducted in compliance with the Declaration of Helsinki and approved by the Institutional Review Board of Asan Medical Center. The requirement for informed consent was waived.

### Histologic evaluation

The entire tumor bed was submitted for pathologic evaluation. Pre-treatment biopsy and surgery specimens were histologically reviewed. The histologic grade of the pre-NAC specimens, and the overall pathologic cancer size (area of the primary tumor bed, including *in situ* carcinoma), and the size of the largest invasive cancer in post-NAC surgery specimens were evaluated. Histologic type was defined according to the WHO criteria, and histologic grade was assessed using the modified Bloom—Richardson classification [22]. pCR was defined as the complete disappearance of invasive cancer cells from breast tissue and lymph nodes(ypT0/Tis, N0). The expression of ER, PR, and HER2 was examined in full sections that were immunostained at the time of diagnosis.

Tumors were classified as HR+/HER2-, HR+/HER2+, HR-/HER2+, or triple-negative (ER-/PR-/HER2-). Tumors were considered HR positive if they contained at least 1% positive nuclei [19]. HER2-positive tumors were defined as those with an immunohistochemistry score of 3+, or as those scoring 2+ or 1+ and showing *HER2* amplification upon fluorescence or silver *in situ* hybridization [23]. The clinicopathologic characteristics of all cases are summarized in Table 1.

**Table 1:** Patient characteristics.

Variable		Cases treated with neoadjuvant chemotherapy (%)
Age	(mean ± standard deviation)	44.3 ± 9.5
Clinical tumor size	≤2cm	30 (5.1)
	2–5cm	381 (64.1)
	>5cm	183 (30.8)
Clinical nodal metastasis	Negative	131 (22.1)
	Positive	463 (77.9)
Histologic type	Invasive carcinoma of no special type	516 (86.9)
	Invasive lobular carcinoma	19 (3.2)
	Micropapillary carcinoma	31 (5.2)
	Mucinous carcinoma	5 (0.8)
	Carcinoma with mucinous differentiation	9 (1.5)
	Metaplastic carcinoma	13 (2.2)
	Tubular carcinoma	1 (0.2)
Hormone receptor status	Negative	247 (41.6)
	Positive	347 (58.4)
HER2 status	Negative	431 (72.6)
	Positive	163 (27.4)
Histologic grade	1	11 (1.9)
	2	398 (67.0)
	3	185 (31.1)

### Assessment of the pathologic response

Responses to NAC were evaluated using six previously reported pathologic response classification systems, including ypTNM stage [22], RCB [15], RDBN [12], Sataloff’s classification [9], Miller—Payne grading [10], and TRR [14] (S1 Material).

### Statistical analysis

The results obtained for each assessment system were analyzed using the Kaplan—Meier method and time-dependent receiver operating characteristic (ROC) curve estimated using inverse probability of censoring weighed (IPCW)[24]. Comparisons of two assessment systems were performed based on the asymptotic Z test [25]. Kappa values were calculated after changing classification categories from 1 to 4 (e.g., Miller—Payne grades 1 and 2 were combined to yield four category values rather than five). Kappa values were interpreted as poor (<0.20), fair (0.21–0.40), moderate (0.41–0.60), good (0.61–0.80), and very good (0.81–1.00). All statistical analyses were performed using SPSS software (version18; SPSS Inc., Chicago, USA) and R program (www.r-project.org). *P*<0.05 was considered significant.

## Results

### Response patterns of each cancer subtype

A pCR was achieved in 4.4% (12/273) of HR+/HER2-, 10.8% (8/74) of HR+/HER2+, 18.0% (16/89) of HR-/HER2+, and 29.7% (47/158) of triple-negative tumors treated with anthracycline with/without taxane-based NAC. However, the response values for each cancer subtype showed a significantly different distribution (Table 2).

**Table 2:** Comparison of pathologic response assessment systems after neoadjuvant chemotherapy for different subtypes of breast cancer. RCB, residual cancer burden; RDBN, residual disease in breast and node; TRR, tumor response ratio.

	Cases treated with neoadjuvant chemotherapy (%)				
	HR+/HER2-	HR+/HER2+	HR-/HER2+	Triple-negative	*P* value
ypTNM Stage					<0.001
0	12 (4.4)	8 (10.8)	17 (19.1)	47 (29.7)	
1	37 (13.6)	23 (31.1)	24 (27.0)	47 (29.7)	
2	147 (53.8)	29 (39.2)	29 (32.6)	38 (24.1)	
3	77 (28.2)	14 (18.9)	19 (21.3)	26 (16.5)	
RCB					<0.001
0	12 (4.4)	8 (10.8)	17 (19.1)	47 (29.7)	
1	19 (7.0)	14 (18.9)	11 (12.4)	7 (4.4)	
2	157 (57.5)	39 (52.7)	40 (44.9)	76 (48.1)	
3	85 (31.1)	13 (17.6)	21 (23.6)	28 (17.7)	
RDBN					<0.001
1	12 (4.4)	8 (10.8)	17 (19.1)	47 (29.7)	
2	59 (21.6)	19 (25.7)	15 (16.9)	24 (15.2)	
3	140 (51.3)	35 (47.3)	36 (40.4)	59 (37.3)	
4	62 (22.7)	12 (16.2)	21 (23.6)	28 (17.7)	
TRR					<0.001
0	17 (6.2)	12 (16.2)	23 (25.8)	53 (33.5)	
>0–0.4	73 (26.7)	28 (37.8)	34 (38.2)	63 (39.9)	
>0.4–1	144 (52.7)	24 (32.4)	26 (29.2)	32 (20.3)	
>1	39 (14.3)	10 (13.5)	6 (6.7)	10 (6.3)	
Sataloff's T					<0.001
T-A	56 (20.5)	24 (32.4)	40 (44.9)	77 (48.7)	
T-B	117 (42.9)	30 (40.5)	28 (31.5)	51 (32.3)	
T-C	88 (32.2)	17 (23.0)	15 (16.9)	27 (17.1)	
T-D	12 (4.4)	3 (4.1)	6 (6.7)	3 (1.9)	
Sataloff's N					<0.001
N-A	64 (23.4)	26 (35.1)	23 (25.8)	71 (44.9)	
N-B	17 (6.2)	14 (18.9)	23 (25.8)	38 (24.1)	
N-C	81 (29.7)	20 (27.0)	20 (22.5)	15 (9.5)	
N-D	111 (40.7)	14 (18.9)	23 (25.8)	34 (21.5)	
Miller—Payne grade					<0.001
5	16 (5.9)	12 (16.2)	20 (22.5)	52 (32.9)	
4	64 (23.4)	25 (33.8)	26 (29.2)	38 (24.1)	
3	143 (52.4)	26 (35.1)	31 (34.8)	48 (30.4)	
2	38 (13.9)	8 (10.8)	6 (6.7)	17 (10.8)	
1	12 (4.4)	3 (4.1)	6 (6.7)	3 (1.9)	

Kappa values were calculated for each tumor subtype in an attempt to identify agreement among the various pathologic response evaluation systems (Table 3). ypTNM stage, RCB, and RDBN showed moderate to good agreement (kappa value, 0.401–0.791) for all subtypes, and TRR showed fair to moderate agreement with ypTNM stage, RCB, and RDBN. Sataloff’s T classification showed moderate agreement with TRR only for HR+/HER2+ and triple-negative tumors. The Miller—Payne grade showed moderate or high agreement with RCB and TRR for all tumor subtypes except HR+/HER2-.

**Table 3:** Kappa values for the different pathologic response assessment systems after systemic neoadjuvant therapy. RCB, residual cancer burden; RDBN, residual disease in breast and node; TRR, tumor response ratio.

		RCB	RDBN	TRR	Sataloff's T	Sataloff's N	Miller—Payne
HR+/HER2-	ypTNM stage	0.467	0.662	0.373	0.127	0.026	0.235
	RCB		0.401	0.239	0.029	0.148	0.300
	RDBN			0.200	0.088	0.079	0.166
	TRR				0.211	0.048	0.367
	Sataloff's T					-0.033	0.119
	Sataloff's N						0.111
HR+/HER2+	ypTNM stage	0.491	0.590	0.545	0.344	0.272	0.278
	RCB		0.592	0.415	0.129	0.154	0.408
	RDBN			0.410	0.221	0.194	0.330
	TRR				0.432	0.109	0.544
	Sataloff's T					0.086	0.309
	Sataloff's N						0.071
HR-/HER2+	ypTNM stage	0.556	0.603	0.421	0.261	0.298	0.389
	RCB		0.681	0.331	0.168	0.228	0.417
	RDBN			0.268	0.190	0.152	0.389
	TRR				0.383	0.073	0.531
	Sataloff's T					0.053	0.270
	Sataloff's N						0.135
Triple-negative	ypTNM stage	0.446	0.560	0.580	0.460	0.149	0.519
	RCB		0.681	0.305	0.204	0.139	0.440
	RDBN			0.344	0.246	0.088	0.497
	TRR				0.539	-0.037	0.633
	Sataloff's T					-0.029	0.285
	Sataloff's N						0.005

### Survival outcomes for each subtype

**Fig 1:**
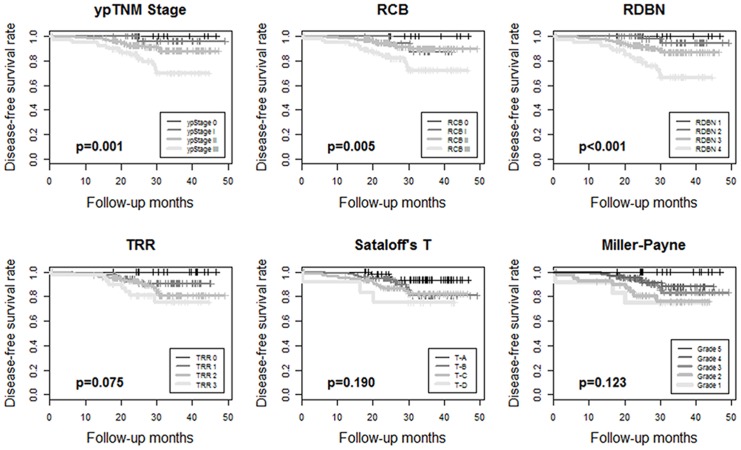
Kaplan—Meier survival curves for ypTNM stage, RCB, RDBN, TRR, Sataloff's T classification, and Miller-Payne grade showing disease-free survival rates for patients with HR+/HER2- breast cancer treated with anthracycline with/without taxane-based neoadjuvant chemotherapy. Three evaluation systems assessing the absolute residual tumor, ypTNM stage, RCB, and RDBN, have prognostic significance. (RCB, residual cancer burden; RDBN, residual disease in breast and node; TRR, tumor response ratio).

**Fig 2:**
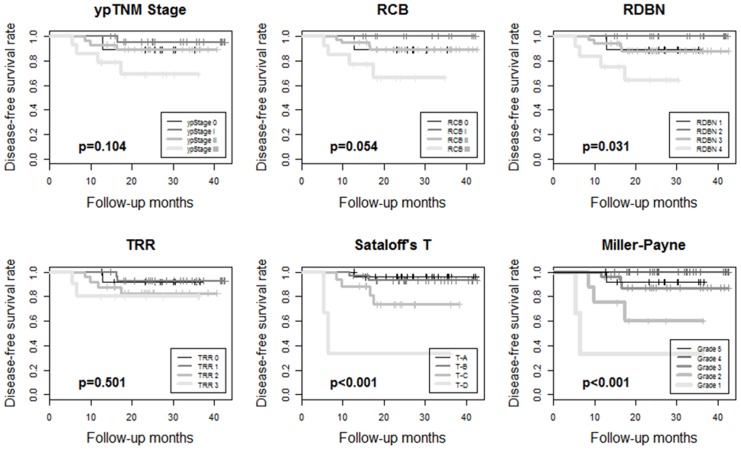
Kaplan-Meier survival curves for ypTNM stage, RCB, RDBN, TRR, Sataloff's T classification, and Miller-Payne grade showing disease-free survival rates for patients with HR+/HER2+ breast cancer treated with anthracycline with/without taxane-based neoadjuvant chemotherapy. None of the evaluation systems yield distinct Kaplan-Meier survival curves, while RDBN, Sataloff's T classification, and Miller-Payne grade show statistical significance (*p*<0.05). (RCB, residual cancer burden; RDBN, residual disease in breast and node; TRR, tumor response ratio).

**Fig 3:**
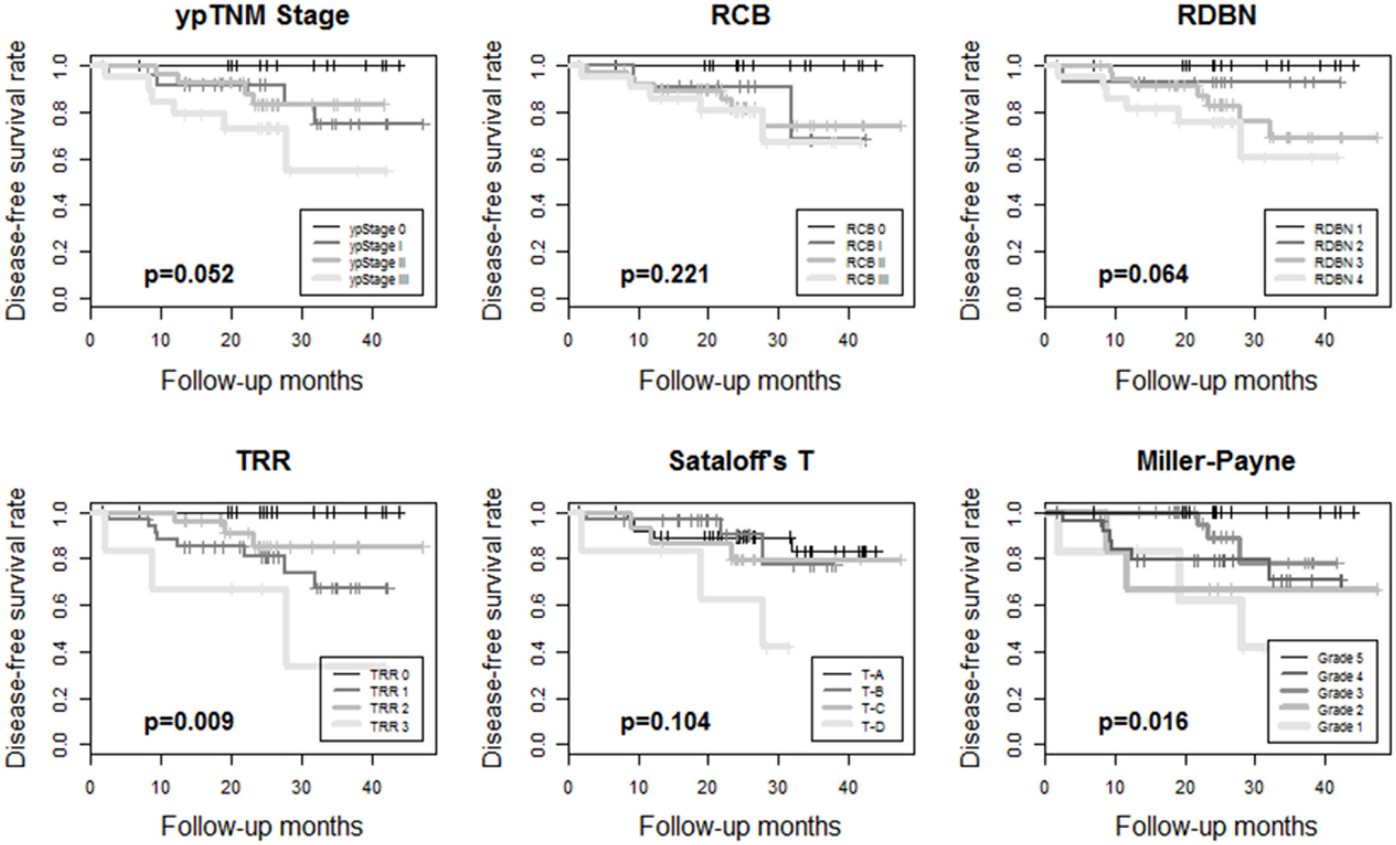
Kaplan-Meier survival curves for ypTNM stage, RCB, RDBN, TRR, Sataloff's T classification, and Miller-Payne grade showing disease-free survival rates for patients with HR-/HER2+ breast cancer treated with anthracycline with/without taxane-based neoadjuvant chemotherapy. None of the evaluation systems yield distinct Kaplan-Meier survival curves, while TRR and Miller-Payne grade show statistical significance (p<0.05). (RCB, residual cancer burden; RDBN, residual disease in breast and node; TRR, tumor response ratio).

**Fig 4:**
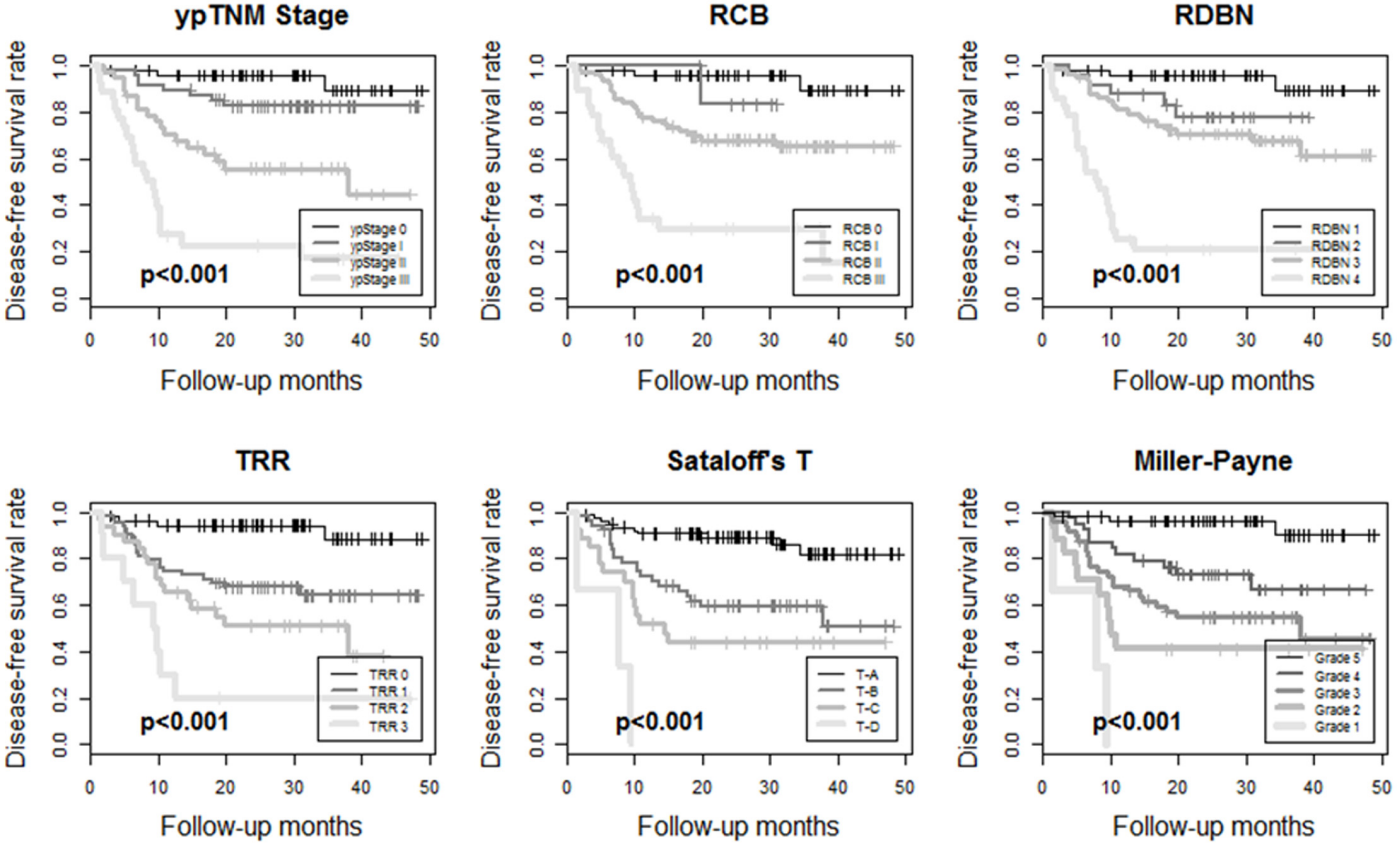
Kaplan-Meier survival curves for ypTNM stage, RCB, RDBN, TRR, Sataloff's T classification, and Miller-Payne grade showing disease-free survival rates for patients with triple negative breast cancer treated with anthracycline with/without taxane-based neoadjuvant chemotherapy. All the pathologic response evaluation systems yield distinct Kaplan-Meier survival curves and have prognostic significance. (RCB, residual cancer burden; RDBN, residual disease in breast and node; TRR, tumor response ratio).

The median follow-up period was 37.2 months. Kaplan-Meier survival analyses showed disease-free survival rates and their prognostic significance for all six pathologic response evaluation systems in each subtype of breast cancers. For HR+/HER2- tumors (Fig 1), systems absolutely assessing the residual tumor (ypTNM stage, RCB, and RDBN) had prognostic significance. For HR+/HER2+ (Fig 2) and HR-/HER2+ tumors (Fig 3), the association between patient survival and pathologic response assessment results varied according to the examination system used. However, none of evaluation systems yielded distinct Kaplan—Meier survival curves for those patients. On the other hand, Kaplan—Meier survival analysis revealed that all of the pathologic response evaluation systems had prognostic significance for triple-negative tumors in terms of disease-free survival (Fig 4). Each evaluation system yielded distinct Kaplan—Meier survival curves for patients with triple-negative breast cancer. Sataloff’s N classification also had prognostic significance for those with HR+/HER2-, HR-/HER+, and triple-negative tumors; however, it only yielded distinct Kaplan—Meier curves for those with triple-negative breast cancer (S1 Fig).

**Fig 5:**
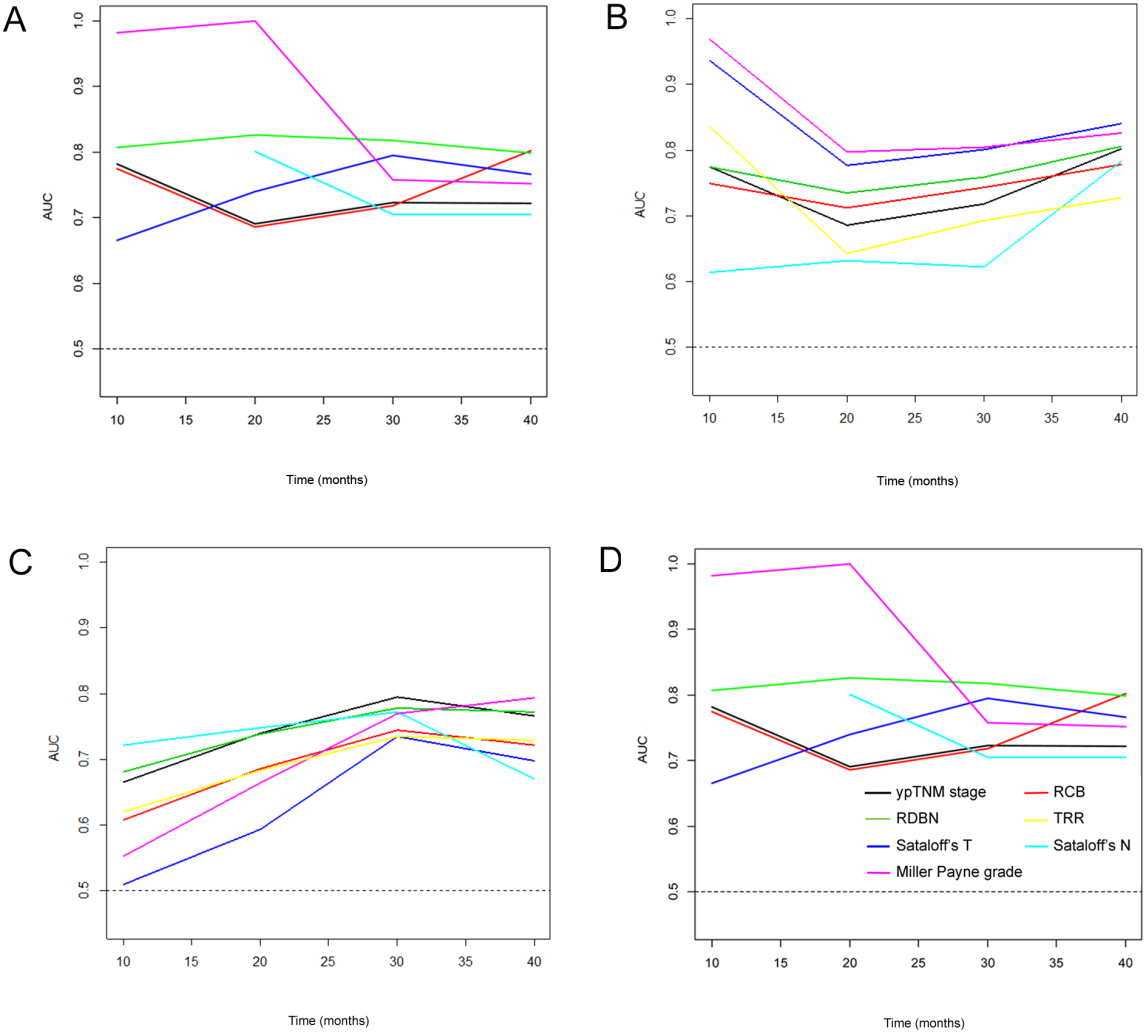
Time-dependent ROC curve estimation analysis for ypTNM stage, RCB, RDBN, TRR, Sataloff's T classification, and Miller-Payne grade in specific subtypes of breast cancer treated with anthracycline with/without taxane-based neoadjuvant chemotherapy. (A) HR+/HER2-. The ranking of predictive accuracy are variably changed through time, while AUC values of all evaluation systems are over 0.5. (B) HR+/HER2+. The rankings of predictive accuracy are variably changed through time, while AUC values of all evaluation systems are over 0.5. (C) HR-/HER2+. The rankings of predictive accuracy are variably changed through time, while AUC values of all evaluation systems are over 0.5. (D) triple-negative tumors. The values of AUC of all evaluation systems are over 0.5 and relatively constant over time (Line color: black, ypTNM stage; red, RCB; green, RDBN; yellow, TRR; blue, Sataloff’s T; sky-blue, Sataloff’s N; pink, Miller Payne grade) (AUC, area under the curve).

To compare the prognostic significance of evaluation systems, time-dependent ROC curve estimation analysis has been performed. In all subtypes, the values of area under the curve (AUC) were over 0.5 in all the assessment systems regardless of time (Fig 5). Only in triple-negative subtype, values of AUC were relatively constant over time. The rankings of predictive accuracy among the systems were variably changed as time passed by. When we compared two evaluation systems among seven systems, none of the evaluation system showed superiority over other systems at every time points (S1 Table).

## Discussion

The present study is the first to examine the prognostic significance of several pathologic response evaluation systems using specimens derived from breast cancer patients undergoing anthracycline with/without taxane-based NAC. We found significant differences in the distribution of response values depending on the subtypes. Kappa values were calculated for each tumor subtype to identify agreement among the various pathologic response evaluation systems. Systems that assessed residual tumor in breast tissue and lymph nodes (ypTNM stage, RCB, and RDBN) showed moderate to good agreement for all tumor subtypes. These three systems also showed fair to moderate agreement with the TRR because the size of the residual tumor in the breast also forms part of the TRR. However, the kappa values for the absolute and relative response evaluation systems were generally lower for HR+/HER2- tumors and higher for triple-negative tumors. This difference may be due to intrinsic differences in the morphology of these two tumor types. Triple-negative breast cancers usually have pushing margins and high cellularity, and tend to shrink in response to NAC without a large reduction in tumor cellularity, resulting in more compact tumors [21]. Therefore, a reduction in size is the main outcome measure of a tumor’s response to NAC. These characteristics of triple-negative tumors may explain why we found better agreement between the absolute and relative response evaluation systems in such cases. Conversely, HR+/HER2- tumors usually show an infiltrative growth pattern and a therapeutic response in a relatively large area of the tumor bed, accompanied by a reduction in cellularity. Thus, tumors that remain large but show reduced overall cellularity may be more common than with triple-negative breast cancer. These features might contribute to the generally lower kappa values calculated for HR+/HER2- tumors between the absolute and relative response evaluation systems.

Although both absolute assessment of the amount of residual tumor and relative assessment of treatment responses (i.e., comparing post-NAC specimens with pre-NAC images or specimens) predict similar clinical outcomes for patients with triple-negative tumors, using absolute assessment systems might be more effective in routine practice. This is because pre-NAC images or biopsy specimens are not always available in a clinical setting; therefore, obtaining results using relative assessment systems might be difficult. Also in HR+/HER2- tumors, systems absolutely assessing the residual tumor (ypTNM stage, RCB, and RDBN) showed prognostic significance. Therefore, absolute response assessment systems appear superior in terms of availability for pathologists and predicting the prognosis of patients with triple-negative and HR+/HER2- tumors after NAC based on anthracycline with/without taxane.

Even though the Miller—Payne grade and the TRR showed prognostic significance in some tumor types, neither system takes lymph node status into account. However, several studies show that integrating lymph node status is important [5, 26, 27]. Similarly, we could find different survival outcome even in tumors with no metastatic tumor cells in lymph nodes according to the presence or absence of response of pre-existing tumor cells. The difference in survival outcome was particularly significant for those with triple-negative breast cancer. Therefore, additional prognostic information may be acquired if pathologic reports mentioned the presence/absence of a therapeutic response in the lymph nodes after anthracycline with/without taxane-based NAC.

Despite of its originality and novelty, this study has some limitations. First, the follow-up period was relatively short, and the number of patients with HER2+ tumors was small. Therefore, further studies with a larger cohort and longer clinical follow-up are warranted. Second, our conclusions are limited to the cases of anthracycline with/without taxane-based NAC. In cases treated with other regimens might show different results, so that further studies including NAC regimens other than anthracycline with/without taxane as well as other neoadjuvant anti-HER2 or hormonal treatments are also warranted.

In conclusion, most of the currently available pathologic assessment systems used after anthracycline with/without taxane-based NAC effectively classified triple-negative breast cancers into groups showing different prognoses. The pathologic assessment systems evaluating residual tumors only also had prognostic significance in HR+/HER2- tumors. However, new assessment methods are required to effectively evaluate the pathologic responses of HR+/HER2+ and HR-/HER2+ tumors to NAC, especially based on anthracycline with/without taxane.

## Supporting Information

S1 Fig Kaplan—Meier survival curves showing disease-free survival according to Sataloff’s N classification. (TIF)

S1 Material Several classification systems assessing responses to neoadjuvant chemotherapy in breast cancers. Among the six evaluation systems used in this study, residual cancer burden (RCB), residual disease in breast and nodes (RDBN), Sataloff’s classification, Miller—Payne grading, and tumor response ratio (TRR) but yp TNM stage were defined in this material.
(DOCX)

S2 Material Clinicohistopathologic information of the patients included in this study. Anonymized data including subtype of breast cancers, the regimen of chemotherapy, and other information essential for the pathologic evaluation systems.
(XLSX)

S1 Table Comparison of area under the curve of pathologic response assessment systems in each subtype at each time point (*P* values). (DOCX)
