# Supplementary figures and images for: Comparison of Pathologic Response Evaluation Systems after Anthracycline with/without Taxane-Based Neoadjuvant Chemotherapy among Different Subtypes of Breast Cancers
Source: PLoS One. 2015 Sep 22;10(9):e0137885. doi: 10.1371/journal.pone.0137885 (PMC4578929; doi:10.1371/journal.pone.0137885)

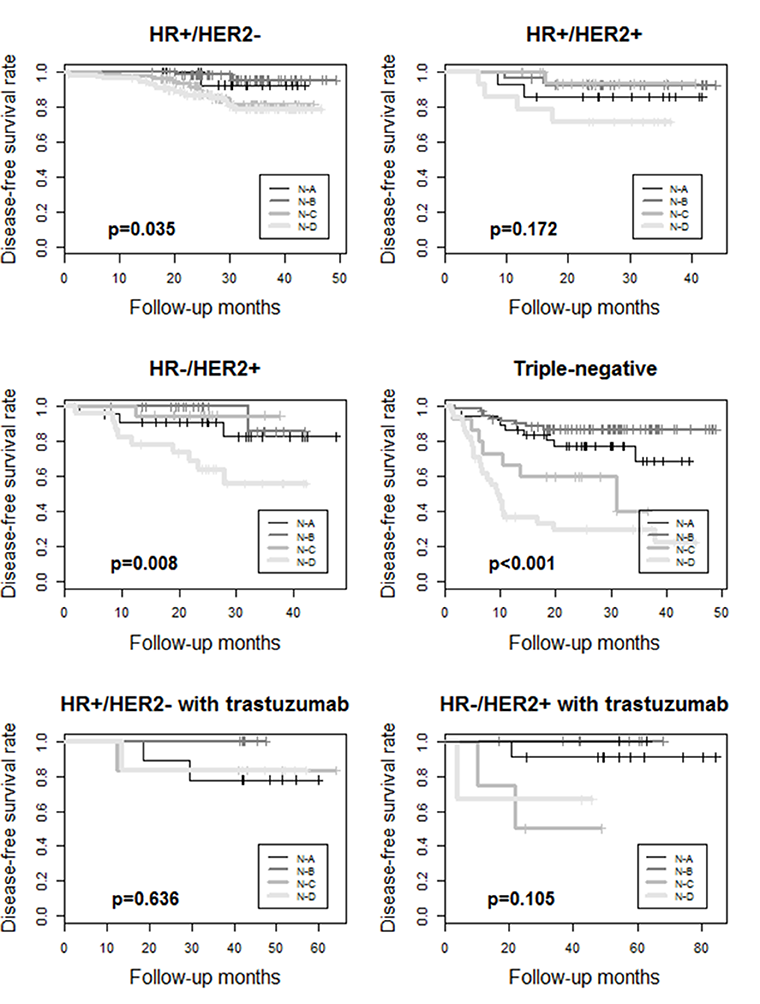

Supplement: S1 Fig — (TIF) [file pone.0137885.s001.tif]
