# Supplementary material for: Comparison of Pathologic Response Evaluation Systems after Anthracycline with/without Taxane-Based Neoadjuvant Chemotherapy among Different Subtypes of Breast Cancers
Source: PLoS One. 2015 Sep 22;10(9):e0137885. doi: 10.1371/journal.pone.0137885 (PMC4578929; doi:10.1371/journal.pone.0137885)
Supplement: S1 Material — Among the six evaluation systems used in this study, residual cancer burden (RCB), residual disease in breast and nodes (RDBN), Sataloff’s classification, Miller—Payne grading, and tumor response ratio (TRR) but yp TNM stage were defined in this material. (DOCX) [file pone.0137885.s002.docx]

RCB

The RCB is calculated as follows: *RCB* = 1.4 (*finvdprim*)^0.17^ + [4(1 - 0.75*^LN^*)*dmet*]^0.17^

where *d_prim_* is derived from the bidimensional diameters of the primary tumor bed in the resected specimen, *f_inv_* is the proportion of the primary tumor bed that contains invasive carcinoma, *LN* is the number of axillary lymph nodes containing metastatic carcinoma, and *d_met_* is the diameter of the largest metastasis in an axillary lymph node.

The cut-off points are 1.36 and 3.28.

RDBN

Level 1: pCR in breast and nodes with or without *in situ* carcinoma

Levels 2–4: Residual disease, calculated as 0.2 (residual breast tumor size in cm) + index of involved nodes (0 for no positive nodes, 1 for 1–4 positive nodes, 2 for 5–7 positive nodes, and 3 for 8 positive nodes) + the Scarff–Bloom–Richardson grade (1, 2, or 3). The cut-off points are 3 and 4.3.

Sataloff’s classification

Breast:

T-A: Total or nearly total therapeutic effect

T-B: >50% therapeutic effect

T-C: <50% therapeutic effect

T-D: No therapeutic effect

Lymph node:

N-A: Therapeutic effect but no metastasis

N-B: No metastasis, no therapeutic effect

N-C: Therapeutic effect but metastasis

N-D: Metastasis, no therapeutic effect

Miller–Payne grading

Grade 1: No change or some changes to individual malignant cells, but no reduction in overall cellularity

Grade 2: Minor loss of tumor cells (up to 30%), but overall cellularity still high

Grade 3: An estimated 30–90% reduction in the number of tumor cells

Grade 4: Marked disappearance of tumor cells such that only small clusters or widely dispersed individual cells remain (loss of >90% of tumor cells)

Grade 5: No invasive cancer cells identifiable in sections from the site of the tumor (carcinoma in situ may be present)

TRR

Calculated as follows: Residual breast disease observed upon pathologic examination divided by the size of the tumor on the pre-NAC image.

TRR = 0 (pCR)

TRR >0–0.4 (strong partial response)

TRR >0.4–1.0 (weak partial response, WPR)

TRR >1.0 (tumor growth)
