# Supplementary material for: Comparison of Pathologic Response Evaluation Systems after Anthracycline with/without Taxane-Based Neoadjuvant Chemotherapy among Different Subtypes of Breast Cancers
Source: PLoS One. 2015 Sep 22;10(9):e0137885. doi: 10.1371/journal.pone.0137885 (PMC4578929; doi:10.1371/journal.pone.0137885)
Supplement: S1 Table — (DOCX) [file pone.0137885.s004.docx]

**S1 Table.** **Comparison of area under the curve of pathologic response assessment systems in each subtype at each time point** (*P* values).

| **Subtypes** | **Compared systems** | **Time (months)** | | | |
| --- | --- | --- | --- | --- | --- |
|  |  | **10** | **20** | **30** | **40** |
| HR+/HER2- | ypTNM stage vs. RCB | 0.163 | 0.993 | 0.651 | 0.186 |
|  | ypTNM stage vs. RDBN | 0.570 | 0.571 | 0.466 | 0.499 |
|  | ypTNM stage vs. TRR | 0.125 | 0.162 | 0.230 | 0.543 |
|  | ypTNM stage vs. Sataloff's T | 0.857 | 0.486 | 0.052 | **0.029** |
|  | ypTNM stage vs. Sataloff's N | 0.649 | 0.660 | 0.148 | **0.019** |
|  | ypTNM stage vs. Miller-Payne | 0.557 | 0.410 | 0.119 | 0.069 |
|  | RCB vs. RDBN | 0.810 | 0.626 | 0.272 | **0.037** |
|  | RCB vs. TRR | 0.183 | 0.163 | 0.331 | 0.667 |
|  | RCB vs. Sataloff's T | 0.996 | 0.429 | **0.042** | 0.170 |
|  | RCB vs. Sataloff's N | 0.805 | 0.607 | 0.184 | 0.107 |
|  | RCB vs. Miller-Payne | 0.449 | 0.317 | 0.115 | 0.332 |
|  | RDBN vs. TRR | **0.049** | **0.020** | 0.074 | 0.315 |
|  | RDBN vs. Sataloff's T | 0.795 | 0.168 | **0.003** | **0.003** |
|  | RDBN vs. Sataloff's N | 0.874 | 0.906 | **0.041** | **0.001** |
|  | RDBN vs. Miller-Payne | 0.115 | 0.187 | **0.018** | **0.010** |
|  | TRR vs. Sataloff's T | **< 0.001** | 0.419 | 0.232 | **0.048** |
|  | TRR vs. Sataloff's N | **0.024** | **0.046** | 0.758 | 0.097 |
|  | TRR vs. Miller-Payne | **< 0.001** | 0.609 | 0.590 | 0.155 |
|  | Sataloff's T vs. Sataloff's N | 0.468 | 0.128 | 0.547 | 0.794 |
|  | Sataloff's T vs. Miller-Payne | **< 0.001** | 0.809 | 0.283 | 0.378 |
|  | Sataloff's N vs. Miller-Payne | **< 0.001** | 0.132 | 0.902 | 0.478 |
| HR+/HER2+ | ypTNM stage vs. RCB | 0.433 | 0.560 | 0.650 | 0.703 |
|  | ypTNM stage vs. RDBN | 1.000 | 0.277 | 0.417 | 0.955 |
|  | ypTNM stage vs. TRR | 0.067 | 0.430 | 0.665 | 0.192 |
|  | ypTNM stage vs. Sataloff's T | **0.002** | 0.293 | 0.342 | 0.671 |
|  | ypTNM stage vs. Sataloff's N | 0.101 | 0.497 | 0.287 | 0.866 |
|  | ypTNM stage vs. Miller-Payne | **0.003** | 0.088 | 0.204 | 0.693 |
|  | RCB vs. RDBN | 0.338 | 0.402 | 0.569 | 0.323 |
|  | RCB vs. TRR | **0.043** | 0.219 | 0.424 | 0.500 |
|  | RCB vs. Sataloff's T | **0.004** | 0.344 | 0.400 | 0.328 |
|  | RCB vs. Sataloff's N | 0.116 | 0.370 | 0.240 | 0.966 |
|  | RCB vs. Miller-Payne | **0.006** | 0.140 | 0.294 | 0.319 |
|  | RDBN vs. TRR | 0.090 | 0.116 | 0.286 | 0.287 |
|  | RDBN vs. Sataloff's T | **0.005** | 0.524 | 0.533 | 0.582 |
|  | RDBN vs. Sataloff's N | 0.083 | 0.267 | 0.192 | 0.861 |
|  | RDBN vs. Miller-Payne | **0.007** | 0.243 | 0.421 | 0.690 |
|  | TRR vs. Sataloff's T | **0.004** | **0.030** | 0.084 | 0.177 |
|  | TRR vs. Sataloff's N | 0.053 | 0.918 | 0.548 | 0.667 |
|  | TRR vs. Miller-Payne | **0.006** | **0.003** | **0.032** | 0.146 |
|  | Sataloff's T vs. Sataloff's N | **0.017** | 0.264 | 0.189 | 0.695 |
|  | Sataloff's T vs. Miller-Payne | 0.092 | 0.576 | 0.921 | 0.742 |
|  | Sataloff's N vs. Miller-Payne | **0.018** | 0.174 | 0.162 | 0.763 |
| HR-/HER2+ | ypTNM stage vs. RCB | 0.418 | 0.280 | 0.343 | 0.582 |
|  | ypTNM stage vs. RDBN | 0.744 | 0.997 | 0.758 | 0.962 |
|  | ypTNM stage vs. TRR | 0.615 | 0.386 | 0.332 | 0.780 |
|  | ypTNM stage vs. Sataloff's T | 0.232 | 0.125 | 0.469 | 0.578 |
|  | ypTNM stage vs. Sataloff's N | 0.223 | 0.805 | 0.481 | 0.193 |
|  | ypTNM stage vs. Miller-Payne | 0.239 | 0.278 | 0.742 | 0.825 |
|  | RCB vs. RDBN | 0.397 | 0.338 | 0.427 | 0.474 |
|  | RCB vs. TRR | 0.826 | 0.959 | 0.849 | 0.919 |
|  | RCB vs. Sataloff's T | 0.174 | 0.128 | 0.860 | 0.770 |
|  | RCB vs. Sataloff's N | 0.150 | 0.273 | 0.639 | 0.596 |
|  | RCB vs. Miller-Payne | 0.332 | 0.610 | 0.598 | 0.369 |
|  | RDBN vs. TRR | 0.481 | 0.320 | 0.372 | 0.389 |
|  | RDBN vs. Sataloff's T | 0.205 | 0.129 | 0.528 | 0.410 |
|  | RDBN vs. Sataloff's N | 0.540 | 0.849 | 0.917 | 0.423 |
|  | RDBN vs. Miller-Payne | 0.163 | 0.243 | 0.871 | 0.748 |
|  | TRR vs. Sataloff's T | 0.075 | 0.136 | 0.994 | 0.678 |
|  | TRR vs. Sataloff's N | 0.331 | 0.368 | 0.574 | 0.676 |
|  | TRR vs. Miller-Payne | **0.001** | 0.574 | 0.471 | 0.178 |
|  | Sataloff's T vs. Sataloff's N | 0.139 | 0.141 | 0.654 | 0.801 |
|  | Sataloff's T vs. Miller-Payne | 0.457 | 0.097 | 0.289 | 0.052 |
|  | Sataloff's N vs. Miller-Payne | 0.125 | 0.291 | 0.969 | 0.349 |
| Triple-negative | ypTNM stage vs. RCB | 0.321 | **0.046** | 0.104 | 0.509 |
|  | ypTNM stage vs. RDBN | 0.946 | 0.430 | 0.391 | 0.092 |
|  | ypTNM stage vs. TRR | **0.023** | 0.097 | 0.136 | 0.372 |
|  | ypTNM stage vs. Sataloff's T | 0.141 | 0.083 | 0.167 | 0.476 |
|  | ypTNM stage vs. Sataloff's N | 0.509 | 0.107 | 0.117 | 0.513 |
|  | ypTNM stage vs. Miller-Payne | 0.357 | 0.158 | 0.199 | 0.395 |
|  | RCB vs. RDBN | 0.334 | 0.218 | 0.426 | 0.618 |
|  | RCB vs. TRR | **0.041** | 0.758 | 0.681 | 0.672 |
|  | RCB vs. Sataloff's T | 0.238 | 0.525 | 0.681 | 0.724 |
|  | RCB vs. Sataloff's N | 0.991 | 0.856 | 0.795 | 0.944 |
|  | RCB vs. Miller-Payne | 0.821 | 0.885 | 0.926 | 0.607 |
|  | RDBN vs. TRR | **0.007** | 0.257 | 0.359 | 0.934 |
|  | RDBN vs. Sataloff's T | 0.071 | 0.184 | 0.383 | 0.958 |
|  | RDBN vs. Sataloff's N | 0.609 | 0.378 | 0.467 | 0.796 |
|  | RDBN vs. Miller-Payne | 0.297 | 0.422 | 0.496 | 0.969 |
|  | TRR vs. Sataloff's T | 0.358 | 0.687 | 0.992 | 0.975 |
|  | TRR vs. Sataloff's N | 0.253 | 0.974 | 0.966 | 0.877 |
|  | TRR vs. Miller-Payne | **0.019** | 0.550 | 0.664 | 0.835 |
|  | Sataloff's T vs. Sataloff's N | 0.493 | 0.828 | 0.962 | 0.883 |
|  | Sataloff's T vs. Miller-Payne | 0.091 | 0.252 | 0.621 | 0.821 |
|  | Sataloff's N vs. Miller-Payne | 0.901 | 0.825 | 0.884 | 0.836 |
